# Performance Characterization of a Plastic Scintillator Sensor for Fast Neutron, Thermal Neutron, and Gamma Ray Discrimination

**DOI:** 10.3390/s26113462

**Published:** 2026-05-30

**Authors:** Yuhang Liu, Fengpeng An, Guang Luo, Wei Wang, Xuesong Zhang, Dixiao Lu, Xiaohao Yin

**Affiliations:** 1 School of Physics, Sun Yat-sen University, No. 135 West Xingang Road, Guangzhou 510275, China; liuyh367@mail2.sysu.edu.cn (Y.L.); zhangxs5@mail2.sysu.edu.cn (X.Z.); ludx6@mail2.sysu.edu.cn (D.L.); yinxh8@mail2.sysu.edu.cn (X.Y.); 2 School of Science, Sun Yat-sen University, No. 66 Gongchang Road, Shenzhen 518107, China; 3 Institut Franco-Chinois de l’Énergie Nucléaire, Sun Yat-sen University, No. 2 Daxue Road, Zhuhai 519082, China

**Keywords:** scintillator, pulse shape discrimination, neutron detector

## Abstract

A compact single-PMT composite scintillation detector was developed and characterized for discrimination of γ rays, fast neutrons, and thermal neutrons in mixed radiation fields. The detector assemblies consisted of either EJ200 or EJ276 plastic scintillators optically coupled to an EJ426 thermal neutron screen and read out by a single photomultiplier tube (PMT). The γ−equivalent energy response of the detector assemblies was calibrated using ^137^Cs, ^22^Na, and ^60^Co sources through Compton edge analysis, and pulse shape discrimination was evaluated with an Am–Be neutron source under different moderator thicknesses. The EJ200+EJ426 assembly provides a well-separated discrimination between thermal neutron capture events and γ dominated events over the measured range, with a figure of merit greater than 5. In contrast, the EJ276+EJ426 assembly produced three identifiable signal populations associated with γ rays, fast neutrons, and thermal neutrons. These results show that the proposed sensor architecture is a promising compact approach for mixed-field radiation applications.

## 1. Introduction

Discrimination of γ rays, fast neutrons, and thermal neutrons in mixed radiation fields is important for radiation monitoring, reactor-related measurements, shielding assessment, nuclear security, and background suppression in nuclear experiments [1,2,3,4]. In such fields, γ rays mainly produce electron-recoil signals, fast neutrons are commonly detected through elastic scattering with hydrogen nuclei in organic scintillators, and thermal neutrons are usually detected through neutron-capture reactions such as Li6(n,α)3H [5,6,7]. Because these radiation components can coexist and may generate overlapping pulse height spectra, reliable event-by-event discrimination is required for quantitative measurements and for suppressing undesired backgrounds [5,8].

Several detector technologies have been developed for neutron/γ discrimination. Liquid organic scintillators can provide good fast neutron/γ pulse shape discrimination (PSD), but their practical use may be limited by flammability, toxicity, leakage risk, and mechanical constraints in compact or field-deployable systems [5,9]. Inorganic scintillators and ^6^Li- or ^10^B-loaded detectors can be effective for thermal neutron detection, but simultaneous identification of γ rays, fast neutrons, and thermal neutrons often requires composite detector structures, dedicated neutron conversion layers, complex optical coupling, or multiple readout channels [10,11,12]. Conventional plastic scintillators are attractive because they are solid, robust, fast, and easy to machine; however, standard plastic scintillators generally have limited intrinsic capability for fast neutron/γ PSD [9,13]. Therefore, a remaining challenge is to develop a compact and mechanically simple detector architecture that can distinguish thermal neutrons from γ rays and, when required, further separate fast neutrons from γ rays using a single photosensor readout.

Hybrid scintillation detectors provide a practical route toward this goal by combining scintillators with complementary response mechanisms and decay characteristics [10,11,12]. In a phoswich-like configuration, different scintillation components are optically coupled to a common photodetector, and their signals are separated through pulse shape analysis [10,14]. Coupling a plastic scintillator to a thermal neutron-sensitive screen is particularly attractive because the plastic scintillator can provide a prompt response to γ rays and fast neutrons, whereas the neutron screen can produce a slower scintillation signal associated with thermal neutron capture [7,11,12,15]. Nevertheless, the discrimination performance of such composite systems depends strongly on the selected plastic scintillator, the neutron conversion screen, the optical coupling, and the signal-processing method [9,12]. In particular, systematic comparisons between a conventional plastic scintillator coupled to a ^6^LiF/ZnS(Ag) screen and a PSD-capable plastic scintillator coupled to the same type of screen are still needed to clarify the specific role of each scintillation component in mixed-field discrimination.

Recent progress in solid organic scintillators has made PSD-capable plastic scintillators promising alternatives to liquid scintillators. Among them, EJ-276 has attracted considerable attention because it combines the practical advantages of plastic scintillators with fast neutron/γ separation capability [13,16,17]. Previous studies have reported the γ ray calibration, response functions, proton light output, and fast neutron detection performance of EJ-276, indicating that it is a suitable candidate for compact mixed-field detectors [18]. In this work, EJ-276 was selected because its radiation-dependent pulse decay characteristics allow fast neutron-induced proton-recoil events to be separated from γ ray induced electron-recoil events by PSD.

In contrast, EJ-200 is a widely used conventional plastic scintillator with high light output, a short decay time, and an emission wavelength compatible with common bialkali photomultiplier tubes [19]. However, EJ-200 does not provide strong intrinsic fast neutron/γ PSD [9,13]. Therefore, EJ-200 was selected as a reference scintillator for evaluating the thermal neutron/γ discrimination capability introduced by the EJ-426 screen without the additional contribution of intrinsic fast neutron/γ PSD.

For thermal neutron detection, ^6^LiF/ZnS(Ag)-based screens such as EJ-426 provide an efficient conversion mechanism through the Li6(n,α)3H reaction and exhibit relatively low γ sensitivity in thin-screen configurations, making them attractive as thermal neutron-sensitive layers in composite detectors [6,7,20,21,22,23]. The slow scintillation component of ZnS(Ag) also provides a temporal signature that can be separated from the faster plastic-scintillator response [6,7,20]. Therefore, EJ-426 was selected as the thermal neutron-sensitive component in the two composite detector assemblies.

In this work, EJ-200, EJ-276, and the ^6^LiF/ZnS(Ag)-based EJ-426 screen were used to construct two compact detector assemblies, EJ200+EJ426 and EJ276+EJ426, for mixed-field response characterization. These materials were selected to provide complementary functions: EJ-200 provides a prompt conventional plastic-scintillator response and serves as a reference material without the intrinsic fast neutron/γ PSD; EJ-276 provides additional fast neutron/γ PSD capability through its radiation-dependent pulse decay behavior; and EJ-426 provides thermal neutron sensitivity through the Li6(n,α)3H conversion reaction and the slower ZnS(Ag) scintillation response. The purpose of using these two configurations was to separate the effects of the thermal neutron screen and the PSD-capable plastic scintillator. The EJ200+EJ426 assembly was designed to evaluate thermal neutron/γ discrimination in a simple plastic-scintillator/screen configuration, whereas the EJ276+EJ426 assembly was designed to extend the discrimination capability to fast neutrons by using the intrinsic fast neutron/γ PSD response of EJ-276. Both assemblies were optically coupled to a single photomultiplier tube, providing a compact single-readout architecture.

The novelty of this work lies in the comparative characterization of two single-PMT composite plastic-scintillator assemblies under identical γ ray calibration and Am–Be neutron irradiation conditions. By comparing EJ200+EJ426 and EJ276+EJ426, this study clarifies how the choice of plastic scintillator affects the separation of γ rays, fast neutrons, and thermal neutrons in a compact phoswich-like detector. Unlike studies focusing on a single PSD scintillator, a single neutron conversion screen, or the intrinsic PSD capability of plastic scintillators alone [13], this work evaluates the respective contributions of the conventional plastic scintillator, the PSD-capable plastic scintillator, and the ^6^LiF/ZnS(Ag) thermal neutron screen within the same experimental framework. This comparison allows the roles of the plastic scintillator and the thermal neutron-sensitive screen to be assessed under the same calibration, irradiation, moderation, and signal-processing conditions. The γ−equivalent energy response was calibrated using ^137^Cs, ^22^Na, and ^60^Co sources, and the PSD performance was evaluated under different HDPE moderator thicknesses. The resulting figure-of-merit values and event-population changes provide a basis for assessing the applicability and limitations of these detector assemblies for compact mixed radiation field measurements.

## 2. Materials and Methods

### 2.1. Detector Configuration and Materials

**Figure 1:**
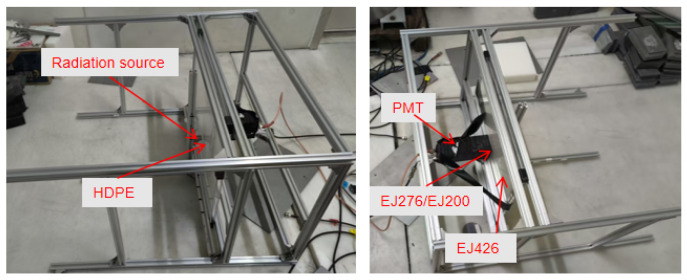
Side view (**left**) and top view (**right**) of the experimental setup. The PMT is wrapped with black tape and coupled with a plastic scintillator.

**Figure 2:**
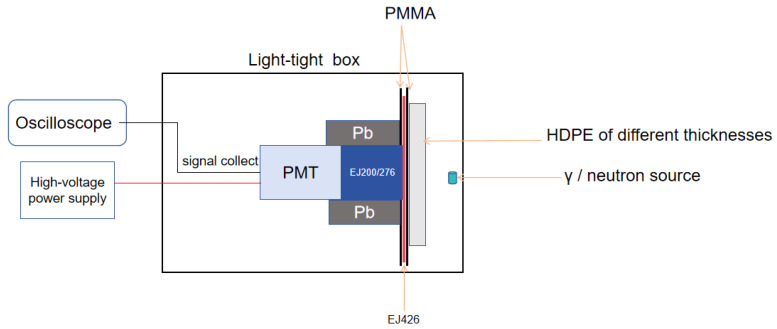
Schematic diagram of the detector configuration. The detector assembly is enclosed in a light-tight box. The plastic scintillator is coupled to the PMT on one side and to the EJ426 neutron screen on the other side. The EJ426 screen is sandwiched between two PMMA plates, one of which has a 6.1 cm × 6.1 cm opening. HDPE layers of different thicknesses are used for neutron moderation in the Am–Be measurements.

Figure 1 presents a photograph of the experimental setup, and Figure 2 shows a schematic diagram of the detector configuration and operating principle. Signals were acquired using a digital oscilloscope (HDO4104A, Teledyne LeCroy, Chestnut Ridge, NY, USA) with a 1 GHz bandwidth and a sampling rate of 10 GS/s [24]. The detector assembly was supported by an external metal frame and enclosed in a light-tight box made of thick black cloth to suppress ambient light interference. Optical coupling between the scintillator and the PMT was achieved using SL612 optical silicone grease (Beijing Hoton Technology Co., Ltd., Beijing, China) [25]. The scintillator modules were wrapped with aluminum foil to improve light collection. Lead bricks were placed around the plastic scintillator to reduce the environmental γ ray background.

Because the EJ426 neutron screen is thin and fragile, it was mechanically protected by sandwiching it between two transparent polymethyl methacrylate (PMMA) plates. A square opening of 6.1 cm × 6.1 cm was machined in the center of one PMMA plate to allow direct coupling between the plastic scintillator and the EJ426 neutron screen. On the source-facing side, the PMMA plate was attached to high-density polyethylene (HDPE) layers of different thicknesses for neutron moderation; this HDPE layer was not used in the γ source experiments. Depending on the measurement purpose, either a neutron source or a γ ray source was placed on the opposite side of the detector assembly.

The three scintillation materials used in this study were EJ200, EJ276, and EJ426, all supplied by Eljen Technology (Sweetwater, TX, USA). These materials were selected to provide complementary scintillation responses for mixed-field measurements. EJ200 served as a conventional fast plastic scintillator and reference material, EJ276 provided fast neutron/γ PSD capability, and EJ426 served as a ^6^LiF/ZnS(Ag)-based thermal neutron-sensitive screen. The basic properties, roles, and reasons for selection of these materials are summarized in Table 1.

As summarized in Table 1, EJ200, EJ276, and EJ426 were selected to provide complementary timing and radiation-response characteristics for comparing the EJ200+EJ426 and EJ276+EJ426 assemblies under similar detector conditions.

Thermal neutrons are captured in EJ426 primarily through the Li6(n,α)3H reaction shown in Equation (1).

(1) Li6+n→H3+He4+4.78 MeV.

This reaction produces ^4^He and ^3^H ions that subsequently excite the ZnS(Ag) phosphor and generate visible photons [6,20]. The resulting light signals from EJ426 exhibit waveform characteristics that differ from the γ induced signals in the plastic scintillator, thereby enabling thermal neutron tagging in the composite detector. Therefore, EJ426 serves as an effective thermal neutron-sensitive layer in mixed radiation fields.

For this experiment, an XP3232 PMT manufactured by Hainan Zhanchuang Photonics Technology Co., Ltd. (Chengmai, Hainan, China) [13] was employed. The XP3232 is a cylindrical vacuum tube with a length of approximately 11.2 cm and a diameter of approximately 5.1 cm. The PMT has a peak spectral sensitivity near 420 nm, which matches the emission spectrum of the plastic scintillators. The photocathode type was bialkali, and the effective photocathode diameter was approximately 5 cm. The PMT was coupled directly to the scintillator.

To improve the reproducibility of the experimental setup, the manufacturers and countries of the main instruments and auxiliary materials used in this work are summarized in Table 2. The scintillation materials are listed separately in Table 1.

### 2.2. PMT Gain Calibration

**Figure 3:**
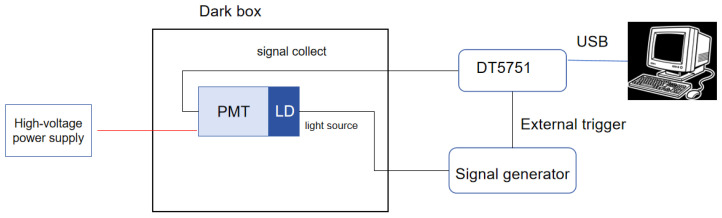
Schematic of the PMT gain calibration setup.

As shown in Figure 3, an experimental system was constructed for PMT gain calibration. The setup consisted of a PMT, a high-voltage power supply (NHR 40 60r, iseg Spezialelektronik GmbH, Radeberg, Germany), a signal generator (AFG3152, Tektronix, Inc., Beaverton, OR, USA), a single-photon light source based on a laser diode (LD; local commercial supplier, Guangzhou, China), and a CAEN DT5751 digitizer module with a bandwidth of 500 MHz and a sampling rate of 1 GS/s [26]. By adjusting the high-voltage power supply, the bias voltage applied to the PMT was precisely controlled, enabling accurate gain calibration.

**Figure 4:**
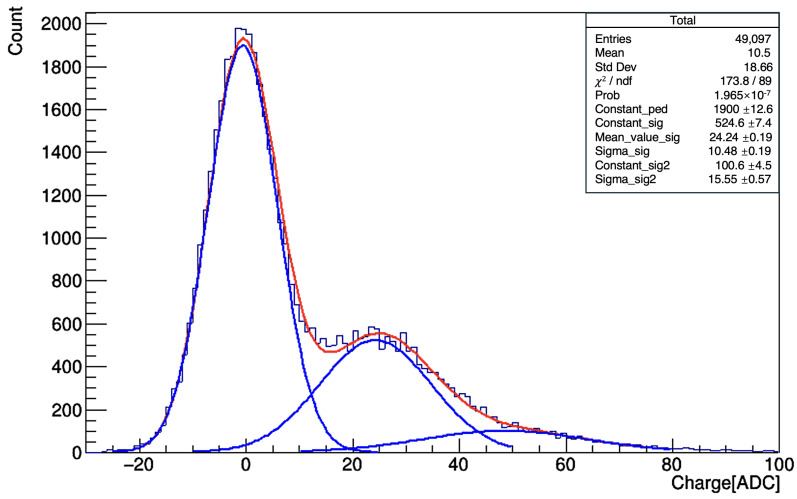
Charge-integrated spectrum of single-photoelectron (SPE) signals measured with the XP3232 PMT. The spectrum was fitted with a sum of Gaussian functions. The red line shows the overall fit to the data using a sum of three Gaussian functions, where the first peak corresponds to the pedestal, the second peak corresponds to the SPE response, and the third peak corresponds to the two-photoelectron component.

The PMT bias voltage was scanned from −1200 to −1400 V in steps of 50 V. For single-photoelectron (SPE) measurements, the settings of the signal generator for driving the LD were first adjusted iteratively while monitoring the PMT output on the CAEN DT5751 digitizer. After repeated tuning, low-amplitude PMT pulses corresponding to the SPE regime were observed. Under these conditions, the signal generator was operated in the pulse mode with continuous output, and the pulse frequency, pulse width, and output amplitude were finally set to 2 kHz, 100 ns, and 1.36 V, respectively. The LD was then used as the light source for SPE calibration. The LD-induced signal pulses were charge-integrated, and the resulting integrals were filled into a histogram. The charge spectrum obtained under these conditions was taken as the SPE spectrum. The integrated for charge SPE spectrum is shown in Figure 4 and was fitted with the sum of three Gaussian functions. Data analysis and fitting were performed using ROOT 6.28/12 (CERN, Geneva, Switzerland).

**Figure 5:**
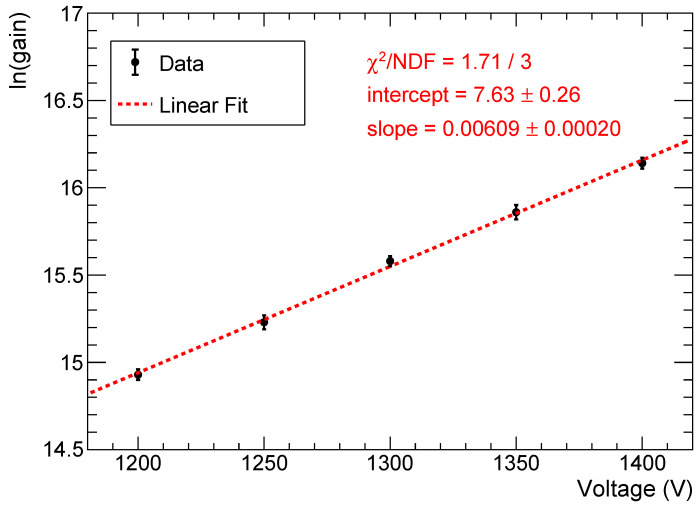
Relationship between the logarithm of PMT gain and applied voltage. The error bars represent the fitting uncertainties of the mean values obtained from Gaussian fits.

The first peak corresponds to the pedestal, the second is the SPE peak, and the third is the two-photoelectron (2PE) peak. After identifying the operating voltage corresponding to the SPE response, the PMT bias voltage was varied stepwise to establish the voltage–gain relationship, as also shown in Figure 5. With the PMT biased at −1200 V, the gain was measured to be approximately $3.0\times 10^{6}$. The corresponding SPE pulse integral was 24.24 in DT5751 channel units, which corresponds to 23.67 mV ns after conversion using a factor of 1000/1024. The integration window used for SPE charge extraction was 100 ns.

### 2.3. Detector Configurations and Measurement Scheme

Two detector configurations were employed in this study.

1. EJ200+EJ426+PMT configuration: This configuration was used for γ response calibration and for evaluating thermal neutron/γ discrimination performance. EJ200 provided the γ−equivalent energy response, whereas EJ426 served as the thermal neutron-sensitive layer.
2. EJ276+EJ426+PMT configuration: This configuration was primarily used under Am–Be irradiation to investigate pulse shape discrimination among fast neutrons, thermal neutrons, and γ rays. EJ276 provided fast neutron/γ discrimination capability, while EJ426 remained sensitive to thermal neutrons. Gamma response calibration and neutron/γ discrimination measurements were performed for both configurations using three γ ray sources (^137^Cs, ^22^Na, and ^60^Co) and one Am–Be neutron source. During the γ response calibration measurements, the activities of the ^137^Cs, ^22^Na, and ^60^Co sources were 1.08 × 10^5^ Bq, 1.02 × 10^5^ Bq, and 3.8 × 10^5^ Bq, respectively. The γ ray sources were positioned at a distance of 10 cm from the source-facing surface of the detector assembly, and the PMT bias voltage and acquisition time were set to −1200 V and 600 s, respectively. The Am–Be neutron source used in this study had a neutron emission rate of approximately $10^{6}$ n/s. During the Am–Be measurements, the source-to-detector distance was 10 cm, and the PMT bias voltage and acquisition time were −1200 V and 600 s, respectively. HDPE moderators with thicknesses ranging from 1 cm to 3 cm were used to obtain different neutron moderation conditions. No Monte Carlo simulation was used to optimize the moderator thicknesses in the present work. The HDPE thicknesses of 1–3 cm were selected experimentally to provide different neutron moderation conditions within the compact source-to-detector geometry and to evaluate the qualitative change in PSD event populations. Therefore, the moderator thickness results are interpreted mainly as relative changes in event composition rather than optimized neutron-transport conditions.

These two configurations were used to assess the mixed-field response of the detector assemblies under the tested conditions.

### 2.4. Gamma Energy Calibration

To enable a consistent comparison of detector responses, a γ ray energy calibration was performed for the EJ200+EJ426 and EJ276+EJ426 assemblies. Three standard γ ray sources, ^137^Cs, ^22^Na, and ^60^Co, were used to provide calibration points over the relevant energy range. The γ ray emission energies were taken from standard nuclear decay data [27]. The corresponding Compton edge energy $E_{C}$ was calculated using the standard Compton scattering relation [5]:

(2) $$E_{C}=E_{\gamma }\left(1-\frac{1}{1+2E_{\gamma }/m_{e}c^{2}}\right)=\frac{2E_{\gamma }^{2}}{m_{e}c^{2}+2E_{\gamma }},$$

where $E_{\gamma }$ is the incident γ ray energy and $m_{e}c^{2}=0.511\;\mathrm{MeV}$ is the electron rest energy. The full-energy γ ray energies and the corresponding calculated Compton edge energies are listed in Table 3.

The scintillators used in this study are organic materials composed primarily of carbon and hydrogen. Owing to their relatively low density and low effective atomic number, their γ ray stopping power is limited, and full-energy absorption peaks are not expected to be prominent in the measured spectra. In the energy range relevant to this study, γ ray interactions in the scintillator are dominated mainly by Compton scattering [5]. Therefore, the γ ray energy calibration was performed using the Compton edges of the measured spectra.

To obtain the gamma response spectra for the two plastic-scintillator assemblies, measurements were performed with the oscilloscope over an acquisition period of 600 s with a threshold of 40 mV. For each source, signals were recorded both with the source present and without the source, and the net energy-response spectrum was obtained by subtracting the background spectrum from the source spectrum.

For ^60^Co, the two γ ray energies are close to each other and could not be resolved separately because of the limited energy resolution of this setup. Therefore, the mean value of the two corresponding Compton edge energies was used as the calibration point for the linear fit.

As described in Section 2.2, the signal integral corresponding to a SPE was measured to be 24.24 in DT5751 channel units, corresponding to 23.67 mV ns, at a PMT bias voltage of −1200 V. The Compton edge positions of the ^137^Cs, ^22^Na, and ^60^Co spectra were determined from the pulse-integrated spectra using a Gaussian-based fit applied to the high-energy edge region. Because full-energy peaks are not prominent in the organic scintillator spectra, the Compton edge region was used for calibration. In the measured pulse integral spectra, the Compton edge appears as a broadened shoulder owing to finite light yield, photoelectron statistics, electronic resolution, and detector-response broadening. Therefore, the Gaussian-based fit was used as an empirical method to determine a reproducible characteristic position in the high-energy edge region. The same fitting procedure was applied to all calibration spectra to ensure a consistent comparison between the two detector assemblies. The fitted peak position μ was then used as the calibration point, and the corresponding fitting uncertainty was taken from the fit. These pulse integral values were subsequently converted into PE counts using the SPE integral. The relationship between PE yield and γ energy was then fitted using Equation (3):

(3) $$N_{\mathrm{PE}}=p_{1}E+p_{0},$$

where $p_{1}$ represents the light yield in PE/MeV and $p_{0}$ accounts for residual offsets introduced by the readout and signal-processing chain. Within the calibrated energy range, both detector assemblies exhibited an approximately linear response.

The measured γ response spectra, fitted Compton edge positions, and linear calibration results are presented in Section 3.1. This calibration relation was subsequently used to convert detector signals into gamma energy for the energy-resolved pulse shape discrimination analysis presented in the following section.

### 2.5. Pulse Shape Discrimination Method

**Figure 6:**
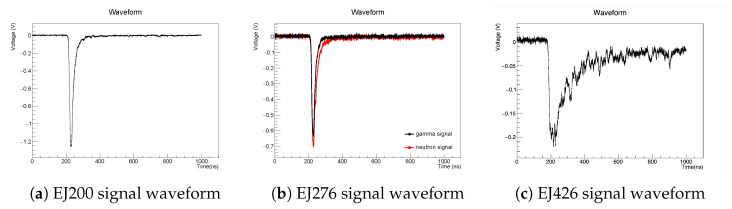
Representative signal waveforms measured from the EJ200, EJ276, and EJ426 scintillators. EJ200 shows a prompt plastic-scintillator response, EJ276 exhibits radiation-dependent pulse decay behavior for fast neutron/γ PSD, and EJ426 shows a slower scintillation component associated with the ZnS(Ag)-based thermal neutron screen.

Pulse shape discrimination (PSD) measurements were performed using the EJ200+EJ426 and EJ276+EJ426 detector assemblies under irradiation with the Am–Be neutron source. The recorded waveforms were analyzed offline to distinguish γ ray, fast neutron, and thermal neutron events on the basis of their different scintillation decay characteristics [8,13,17]. Representative signal waveforms of EJ200, EJ276, and EJ426 are shown in Figure 6. EJ200 exhibits a prompt plastic-scintillator response without a clearly distinguishable delayed component for fast neutron/γ PSD, whereas EJ276 shows radiation-dependent pulse decay behavior that enables fast neutron/γ discrimination. In contrast, EJ426 produces a much slower scintillation signal associated with the ZnS(Ag)-based thermal neutron screen. In the composite detector assemblies, the EJ426 screen produced relatively slow pulses associated with thermal neutron capture, whereas the plastic scintillators exhibited faster scintillation responses. In particular, EJ276 enabled pulse shape-based discrimination between fast neutrons and γ rays, while EJ200 mainly contributed to the prompt scintillation signal without intrinsic fast neutron/γ PSD capability.

For each event, the pulse integral was calculated over two different time windows after the trigger using a charge-comparison PSD method [8,13,17]. A short integration gate of 75 ns was used to characterize the prompt component of the signal, whereas a long integration gate of 800 ns was used to represent the total collected charge. These integration windows were selected according to the measured waveform characteristics and were kept fixed in all PSD analyses to ensure a consistent comparison.

The pulse shape parameter was then defined according to Equation (4):

(4) $$PSD=1-\frac{Q_{\mathrm{short}}}{Q_{\mathrm{long}}},$$

where $Q_{\mathrm{short}}$ and $Q_{\mathrm{long}}$ denote the charge integrals obtained within the short and long gates, respectively. Here, $Q_{\mathrm{short}}$ mainly represents the prompt scintillation component, whereas $Q_{\mathrm{long}}$ includes both the prompt and delayed components. Therefore, the quantity $1-Q_{\mathrm{short}}/Q_{\mathrm{long}}$ represents the relative contribution of the delayed component to the total pulse charge. Events with a larger slow component, such as thermal neutron capture signals from EJ426 or neutron-induced pulses in EJ276, are expected to have larger PSD values.

Two-dimensional distributions of PSD versus pulse integral were constructed for the different detector assemblies and moderator conditions. One-dimensional projections were then obtained for PSD peak identification and fitting. For the EJ200+EJ426 assembly, the analysis focused mainly on the separation between thermal neutron and γ ray events. For the EJ276+EJ426 assembly, the analysis was extended to the discrimination among fast neutrons, thermal neutrons, and γ rays.

The discrimination performance was quantified using the figure of merit (FOM), defined as [8,17]

(5) $$FOM=\frac{|\mu_{1}-\mu_{2}|}{{\mathrm{FWHM}}_{1}+{\mathrm{FWHM}}_{2}},$$

where $\mu_{1}$ and $\mu_{2}$ are the central positions of the two PSD peaks under consideration, and ${\mathrm{FWHM}}_{1}$ and ${\mathrm{FWHM}}_{2}$ are their full widths at half maximum. For the EJ276+EJ426 assembly, separate FOM values were evaluated for the fast neutron/γ, thermal neutron/γ, and thermal neutron/fast neutron peak pairs whenever the corresponding peaks could be resolved.

To investigate the energy dependence of the discrimination performance, the pulse integral values were converted into gamma energy using the calibration relation established in Section 2.4. It should be noted that the neutron energy was not calibrated; therefore, the energies of the neutron events in the PSD plot are actually equivalent gamma energies. The data were then analyzed within selected gamma energy intervals using 500 keV windows, and the corresponding PSD projections were fitted to evaluate the variation of the FOM with energy. This procedure was used in particular to assess the energy range over which fast neutron/γ discrimination in the EJ276+EJ426 assembly became effective.

For the Am–Be measurements, HDPE moderators with different thicknesses were introduced on the source-facing side of the detector assembly to study the effect of neutron moderation on the PSD distributions. The same PSD procedure was applied to all moderator conditions so that the relative changes in thermal neutron, fast neutron, and γ ray event populations could be compared consistently.

## 3. Results

### 3.1. Energy Calibration Results

**Figure 7:**
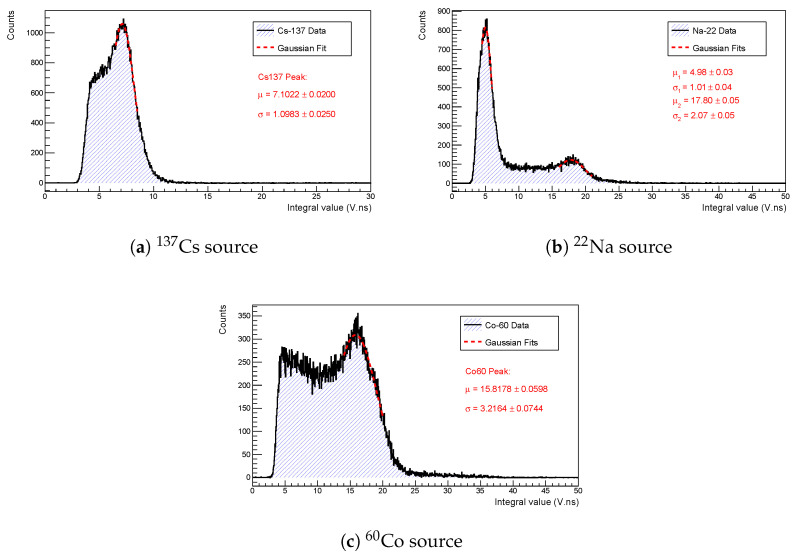
Pulse-integrated gamma response spectra of the EJ200+EJ426 assembly measured with the ^137^Cs, ^22^Na, and ^60^Co sources. The Compton edge regions were fitted to extract the corresponding calibration points.

The gamma response spectra obtained from the EJ200+EJ426 and EJ276+EJ426 assemblies were analyzed using the calibration procedure described in Section 2.4. After background subtraction, the Compton edge regions of the ^137^Cs, ^22^Na, and ^60^Co spectra were identified and fitted to extract the corresponding calibration points. Representative fitted spectra are shown in Figure 7. The same Compton edge fitting procedure was applied to the EJ276+EJ426 assembly.

**Figure 8:**
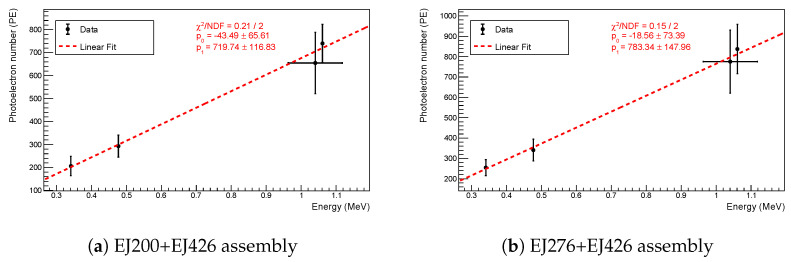
Linear calibration results for the two detector assemblies. (**a**) Relationship between PE yield and γ−equivalent energy for the EJ200+EJ426 assembly. (**b**) Relationship between PE yield and γ−equivalent energy for the EJ276+EJ426 assembly. The data points were obtained from the fitted Compton edge positions, and the solid lines represent the corresponding linear fits.

For both detector assemblies, the pulse integrals were converted into PE yields using the SPE integral determined in Section 2.2, and the resulting PE yields were fitted with a linear function as a function of gamma energy. The measured calibration points and the corresponding linear fits are shown in Figure 8.

Within the calibrated energy range, both detector assemblies exhibited a linear response. This linearity provides the basis for the subsequent energy-resolved PSD analysis. For the EJ200+EJ426 assembly, the fitted parameters were $p_{1}=716.5\pm 114.2$ PE/MeV and $p_{0}=-42.19\pm 64.82$, whereas for the EJ276+EJ426 assembly, the fitted parameters were $p_{1}=781.9\pm 144.5$ PE/MeV and $p_{0}=-17.99\pm 72.34$. The corresponding fitting uncertainties were obtained from the linear fits. The fitted intercepts $p_{0}$ are statistically consistent with zero within their uncertainties. The relatively large uncertainties of $p_{0}$ mainly arise from the limited number of calibration points and the uncertainty associated with the Compton edge position determination. Therefore, the negative fitted $p_{0}$ values should not be interpreted as evidence of detector non-proportionality. Within the calibrated energy range, the detector response is adequately described by the linear relation, and the slope $p_{1}$ was used as the effective light yield for subsequent γ energy conversion.

### 3.2. Pulse Shape Discrimination of the EJ200+EJ426 Assembly

**Figure 9:**
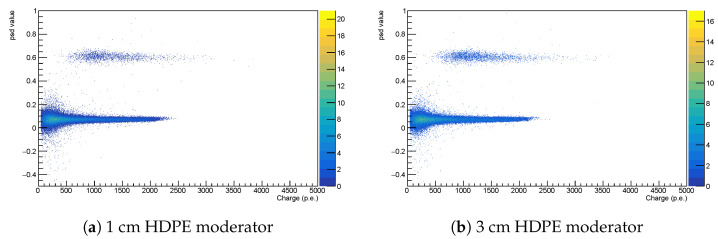
Two-dimensional distributions of PSD value versus charge (PE) for the EJ200+EJ426 assembly under Am–Be irradiation. In both cases, two main event populations can be identified, corresponding predominantly to γ ray induced events and thermal neutron capture events. The relative population of the high-PSD band increases with increasing HDPE thickness.

**Figure 10:**
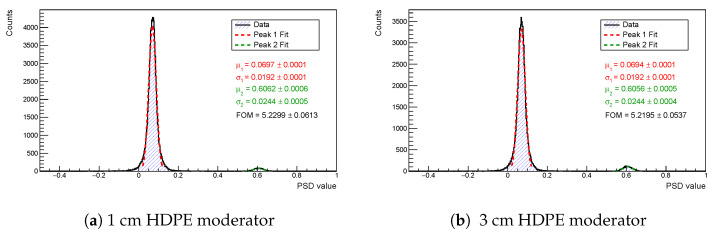
One-dimensional PSD projections and Gaussian fits for the EJ200+EJ426 assembly under Am–Be irradiation. For both HDPE thicknesses shown in the left and right panels, two PSD components were identified and fitted, corresponding mainly to γ ray induced events and thermal neutron capture events. The extracted FOM values indicate effective thermal neutron/γ discrimination under the tested conditions.

The pulse shape discrimination results of the EJ200+EJ426 assembly with different HDPE moderator thicknesses under Am–Be irradiation are shown in Figure 9 and Figure 10. In the two-dimensional distributions of PSD value versus charge, two main event populations can be identified. The low-PSD band is attributed mainly to γ ray induced events in the EJ200 scintillator, whereas the high-PSD band corresponds to thermal neutron capture events associated with the EJ426 screen. As the HDPE moderator thickness increases from 1 cm to 3 cm, the relative population of the high-PSD band increases, indicating enhanced moderation of the neutrons.

To quantify the separation performance, one-dimensional PSD projections were extracted and fitted with two Gaussian functions, as shown in Figure 10. For the 1 cm HDPE condition, the fitted peak positions were $\mu_{1}=0.0697$ and $\mu_{2}=0.6062$, with corresponding widths of $\sigma_{1}=0.0192$ and $\sigma_{2}=0.0244$, yielding an FOM of 5.2299±0.0613. For the 3 cm HDPE condition, the fitted peak positions were $\mu_{1}=0.0694$ and $\mu_{2}=0.6056$, with widths of $\sigma_{1}=0.0192$ and $\sigma_{2}=0.0244$, corresponding to an FOM of 5.2195±0.0537.

The large separation between the two PSD components and the consistently high FOM values confirm that the EJ200+EJ426 assembly provides effective thermal neutron/γ discrimination under the tested conditions.

### 3.3. Pulse Shape Discrimination of the EJ276+EJ426 Assembly

**Figure 11:**
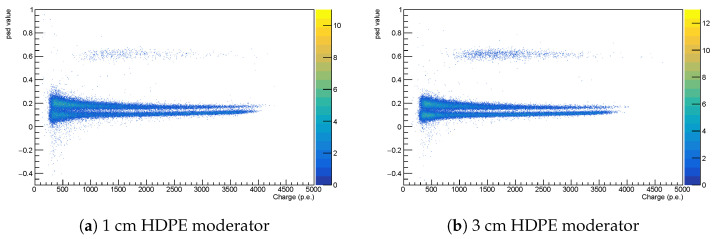
Two-dimensional distributions of PSD value versus charge (PE) for the EJ276+EJ426 assembly under Am–Be irradiation. Left: measurement with a 1 cm HDPE moderator. Right: measurement with a 3 cm HDPE moderator. Three main event populations can be identified, corresponding predominantly to γ ray induced events, fast neutron-induced events, and thermal neutron capture events.

**Figure 12:**
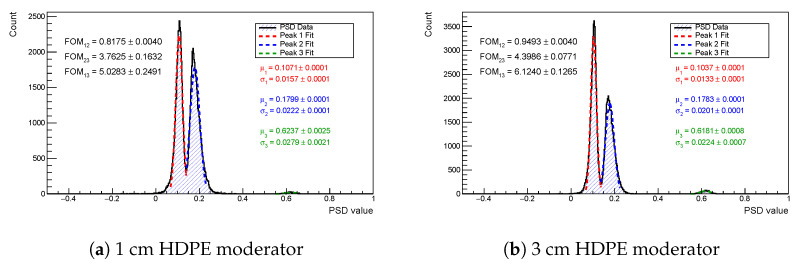
One-dimensional PSD projections and Gaussian fits for the EJ276+EJ426 assembly under Am–Be irradiation. Left: result obtained with a 1 cm HDPE moderator. Right: result obtained with a 3 cm HDPE moderator. Three PSD components were identified and fitted, corresponding mainly to γ ray induced events, fast neutron-induced events, and thermal neutron capture events.

**Figure 13:**
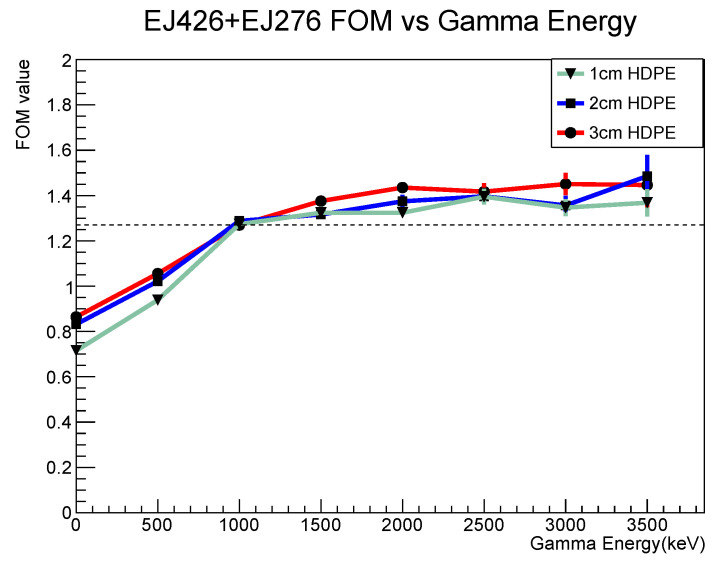
Energy dependence of the fast neutron/γ discrimination performance for the EJ276+EJ426 assembly. The FOM was evaluated in selected γ−equivalent energy intervals under different HDPE moderator thicknesses. The dashed line indicates FOM = 1.27.

The pulse shape discrimination results of the EJ276+EJ426 assembly under Am–Be irradiation are shown in Figure 11, Figure 12 and Figure 13. In the two-dimensional distributions of PSD value versus charge, three main event populations can be identified. The low-PSD band is attributed mainly to γ events in the EJ276 scintillator, the intermediate-PSD band corresponds predominantly to fast neutron events, which are actually the recoiled protons depositing their energy in the scintillator, and the high-PSD band is associated with thermal neutron capture events in the EJ426 screen. Compared with the EJ200+EJ426 assembly, the EJ276+EJ426 configuration exhibits an additional PSD component arising from the intrinsic fast neutron/γ discrimination capability of EJ276.

To quantify the separation performance, one-dimensional PSD projections were extracted and fitted with three Gaussian functions, as shown in Figure 12. The fitted PSD peak positions and the corresponding FOM values for different HDPE moderator thicknesses are summarized in Table 4. The three fitted peaks are associated mainly with γ ray, fast neutron, and thermal neutron events, respectively.

The fitted results show that the separation between the thermal neutron peak and the other two PSD components is pronounced, whereas the fast neutron and γ ray peaks remain partially overlapped in the full-spectrum analysis. In both moderator conditions, the thermal neutron/γ and thermal neutron/fast neutron separations are strong, with FOM values exceeding 3.7, whereas the fast neutron/γ FOM value is below 1.0 in the full-spectrum analysis. These results indicate that the EJ276+EJ426 assembly provides clear thermal neutron tagging and additional fast neutron/γ discrimination capability, although the fast neutron/γ separation is limited when all energies are included.

To investigate the energy dependence of the fast neutron/γ discrimination performance, the pulse integral values were converted into γ−equivalent energy using the calibration relation established in Section 2.4. The resulting FOM variation with gamma energy is shown in Figure 13. As the gamma energy increases, the separation between the fast neutron and γ ray PSD components becomes more pronounced, and the corresponding FOM increases accordingly. In particular, the fast neutron/γ FOM exceeds the commonly used effective-separation criterion of 1.27 (equivalent to a 3σ separation [8,17]) above approximately 1 MeV γ−equivalent energy.

Under the present experimental conditions, the EJ276+EJ426 assembly provides a practical route toward discrimination among fast neutrons, thermal neutrons, and γ rays when the applicable gamma energy range is taken into account. To further examine the effect of neutron moderation, the event populations identified from the PSD analysis were counted for different HDPE moderator thicknesses, as summarized in Table 5. With increasing HDPE thickness, the relative fraction of events classified as thermal neutrons increased from (0.463±0.018)% to (0.568±0.022)%, whereas the relative fraction of fast neutron events decreased from (45.29±0.13)% to (38.99±0.14)%. Meanwhile, the relative fraction of γ ray events increased from (54.25±0.13)% to (60.44±0.14)%. It should be noted that the absolute thermal neutron counts remained statistically comparable within Poisson uncertainties.

These results indicate that increasing the HDPE thickness changes the relative event composition observed with the EJ276+EJ426 assembly. A thicker HDPE moderator enhances neutron moderation, leading to a lower relative fraction of events classified as fast neutrons and a slightly higher relative fraction of events classified as thermal neutrons. However, the absolute thermal neutron counts, such as 674±26 for 1 cm HDPE and 672±26 for 3 cm HDPE, are statistically comparable. Therefore, the moderator thickness dependence should be interpreted mainly as a change in the relative event composition rather than as evidence of a monotonic increase in the absolute thermal neutron detection rate. At larger thicknesses, however, some moderated neutrons may be attenuated within the HDPE through scattering and, to a lesser extent, capture by hydrogen before reaching the detector.

## 4. Discussion

The PSD behavior observed in the two detector assemblies can be understood from the different scintillation mechanisms and decay characteristics of the three scintillation components. In the EJ200+EJ426 assembly, EJ200 provides a prompt plastic-scintillator response to γ rays and recoil charged particles, whereas the EJ426 screen produces a slower scintillation signal associated with thermal neutron capture in the ^6^LiF/ZnS(Ag) layer. The large difference between the prompt EJ200 response and the slower ZnS(Ag) response is the main reason why the thermal neutron events and γ dominated events form two well-separated PSD populations. In contrast, EJ200 itself does not provide strong intrinsic fast neutron/γ PSD because the pulse shape difference between electron-recoil and proton-recoil events is not sufficiently pronounced in this conventional plastic scintillator. Therefore, the EJ200+EJ426 configuration is mainly suitable for thermal neutron tagging relative to the γ ray background rather than for simultaneous fast neutron/γ separation.

For the EJ200+EJ426 assembly, the high FOM values obtained for the thermal neutron/γ pair indicate that the composite detector effectively separates the slow EJ426 thermal neutron signal from the prompt EJ200 signal. This result confirms the advantage of coupling a fast plastic scintillator to a ^6^LiF/ZnS(Ag)-based thermal neutron screen in a single-readout geometry. The result also shows that even without intrinsic fast neutron/γ PSD in the plastic scintillator, thermal neutron tagging can still be achieved when the neutron conversion layer has a sufficiently different temporal response. From an application perspective, this configuration may be useful when the main objective is to identify thermal neutron capture events in the presence of γ ray background using a compact and simple detector structure.

The EJ276+EJ426 assembly shows a more complex PSD response because two discrimination mechanisms are present simultaneously. First, EJ276 provides fast neutron/γ discrimination through the different pulse decay characteristics of γ ray induced electron recoils and fast neutron-induced proton recoils. Second, EJ426 provides a slower thermal neutron signal through the ^6^Li(n,α)^3^H reaction followed by ZnS(Ag) scintillation. As a result, three event populations can be observed and associated mainly with γ rays, fast neutrons, and thermal neutrons. The pronounced separation between the thermal neutron peak and the other two components originates from the much slower EJ426 signal, whereas the partial overlap between the fast neutron and γ ray components reflects the more subtle difference between proton-recoil and electron-recoil pulses in EJ276.

The energy-dependent FOM analysis further supports this interpretation. At low γ−equivalent energies, the fast neutron/γ separation in EJ276+EJ426 is limited because the number of detected photoelectrons is relatively small and the pulse shape parameter is more strongly affected by statistical fluctuations, electronic noise, and threshold effects. As the deposited energy increases, the photoelectron statistics improve, and the difference between the slow components of neutron-induced and γ induced pulses becomes more distinguishable. Consequently, the fast neutron/γ FOM increases with γ−equivalent energy, and effective separation is achieved mainly above approximately 1 MeV under the present experimental conditions. This behavior indicates that the EJ276+EJ426 assembly is more suitable for fast neutron/γ discrimination in the higher γ−equivalent energy region, while its low-energy discrimination capability remains limited.

The moderator thickness dependence provides additional insight into the response of the composite detector in a mixed neutron field. Increasing the HDPE thickness enhances neutron moderation, which reduces the relative fraction of events classified as fast neutrons and slightly increases the relative fraction of events classified as thermal neutrons. However, the absolute number of thermal neutron events does not increase monotonically with moderator thickness. This behavior can be explained by the competition between neutron moderation and neutron loss in the moderator. While additional HDPE can slow down more fast neutrons, it can also scatter neutrons away from the detector or attenuate part of the neutron flux before the neutrons reach the EJ426 screen. Therefore, the moderator thickness results should be interpreted mainly as changes in the relative event composition rather than as a direct measurement of the absolute thermal neutron detection efficiency.

The comparison between the two detector assemblies clarifies the role of the selected plastic scintillator in the composite design. EJ200+EJ426 provides a simple and effective approach for thermal neutron/γ discrimination, while EJ276+EJ426 extends the discrimination capability to fast neutrons by introducing the intrinsic PSD response of EJ276. Therefore, the two configurations are suitable for different but complementary measurement objectives. The EJ200+EJ426 assembly is more appropriate when compact thermal neutron tagging is the main requirement, whereas the EJ276+EJ426 assembly is more appropriate when discrimination among γ rays, fast neutrons, and thermal neutrons is required and the relevant energy range is considered.

It is also useful to compare the present results with other reported multi-radiation discrimination systems. Sharma et al. demonstrated a composite heterogeneous scintillator for triple pulse shape discrimination and capture-gated spectroscopy by combining a PSD-capable plastic scintillator with a ^6^LiF:ZnS(Ag) neutron-sensitive layer [28]. Their detector was designed to distinguish high-energy photons, fast neutron recoil events, and neutron-capture events using distinct pulse shape features [28]. This work provides an important reference for composite scintillator designs aimed at triple discrimination. Compared with that design, the present study focuses on commercially available EJ200, EJ276, and EJ426 components and directly compares EJ200+EJ426 and EJ276+EJ426 assemblies under the same calibration, irradiation, moderation, and signal-processing conditions.

More recently, Frangville et al. reported the Omniscinti detector, a triple-layered phoswich detector designed for simultaneous detection of alpha, beta, gamma, fast neutron, and thermal neutron radiation [29]. That design demonstrates the feasibility of separating multiple radiation types using layered scintillation materials with different temporal and energy-response characteristics [29]. However, such a multi-layer phoswich system is more complex than the two-component plastic-scintillator/EJ426 assemblies investigated here. Therefore, the present work emphasizes a simpler single-PMT configuration for gamma ray, fast neutron, and thermal neutron discrimination rather than a broader multi-particle identification capability.

Stilbene–^6^Li-glass composite scintillators have also been investigated for triple discrimination of gamma rays, fast neutrons, and thermal neutrons [30]. For example, recent work on a high-speed digital system based on a stilbene–^6^Li glass composite detector demonstrated triple discrimination using PSD analysis and digital waveform acquisition [30]. Compared with stilbene-based or ^6^Li-glass-based systems, the present detector uses plastic scintillators and an EJ426 ^6^LiF/ZnS(Ag) screen, which provides a mechanically simple and commercially accessible scintillator screen configuration.

Compared with Jiang et al., who investigated the PSD power of plastic scintillators and reported thermal neutron/γ discrimination using an EJ200+EJ426 combination as well as fast neutron/γ discrimination using EJ276 and UPS-113NG plastic scintillators [13], the present work further investigates an EJ276+EJ426 composite assembly for identifying gamma ray, fast neutron, and thermal neutron event populations within the same detector configuration. Thus, the main improvement of the present approach is not claimed to be a universally superior FOM or a lower-cost system, but rather the controlled comparison of EJ200+EJ426 and EJ276+EJ426 assemblies and the demonstration that replacing EJ200 with EJ276 extends a compact EJ426-coupled detector from thermal neutron/γ tagging toward three-component mixed-field discrimination.

Several limitations of the present work should be noted. First, the present measurements do not provide absolute detection efficiencies for fast neutrons, thermal neutrons, and γ rays. Second, the neutron energies were not calibrated; therefore, the energy-dependent PSD analysis was performed using γ−equivalent energy rather than true neutron energy. Third, the Am–Be source produces a broad neutron spectrum accompanied by γ rays, so the measured PSD populations represent the detector response under a mixed-field condition rather than monoenergetic neutron response. Finally, the detector geometry, EJ426 screen coupling, moderator thickness, and integration windows were not fully optimized. Future work should include absolute efficiency measurements, Monte Carlo simulations of neutron transport and thermalization, optimization of the scintillator screen geometry, and measurements with calibrated neutron fields to further quantify the detector response.

## 5. Conclusions

In this work, two compact single-PMT composite scintillation detector assemblies, EJ200+EJ426 and EJ276+EJ426, were constructed and compared for mixed-field discrimination of γ rays, fast neutrons, and thermal neutrons. A γ−equivalent energy calibration was established using ^137^Cs, ^22^Na, and ^60^Co sources, and the PSD performance was evaluated under Am–Be irradiation with different HDPE moderator thicknesses.

The EJ200+EJ426 assembly provided effective thermal neutron/γ discrimination by combining the prompt response of EJ200 with the slower thermal neutron capture signal from the ^6^LiF/ZnS(Ag)-based EJ426 screen. The EJ276+EJ426 assembly further extended the discrimination capability to fast neutrons by using the intrinsic fast neutron/γ PSD response of EJ276. The comparison between the two assemblies demonstrates that the choice of plastic scintillator plays an important role in determining the discrimination capability of the composite detector.

The HDPE moderator measurements showed that moderator thickness changes the relative event composition by reducing the fraction of fast neutron events and slightly increasing the fraction of events classified as thermal neutrons. However, the absolute thermal neutron counts remained statistically comparable within counting uncertainties, indicating that the moderator effect should be interpreted mainly as a change in relative event populations rather than as a monotonic increase in the thermal neutron detection rate.

Overall, EJ200+EJ426 is suitable for compact thermal neutron tagging in the presence of γ ray background, whereas EJ276+EJ426 is more suitable for applications requiring discrimination among γ rays, fast neutrons, and thermal neutrons when the applicable γ−equivalent energy range is considered. Future work will focus on absolute efficiency measurements, neutron energy calibration, Monte Carlo simulations of neutron transport and moderation, and optimization of the scintillator screen coupling geometry.

## Figures and Tables

**Table 1:** Basic properties, roles, and reasons for selection of the scintillation materials used in this study.

Material	Geometry	Key Specifications	Role in this Work	Reasons for Selection
EJ200	Cube, 6 cm side length	Emission peak: 425 nm; decay time: 2.1 ns; high light output	Conventional plastic scintillator and reference material	Provides a prompt plastic-scintillator response and serves as a reference without strong intrinsic fast neutron/γ PSD capability.
EJ276	Cube, 6 cm side length	Emission peak: 450 nm; multi-component decay for γ and neutron induced pulses ^a^	PSD-capable plastic scintillator	Provides fast neutron/γ discrimination because the pulse decay behavior differs for γ ray induced electron recoils and fast neutron-induced proton recoils.
EJ426	Screen, 42 cm × 42 cm × 0.32 mm	^6^LiF/ZnS(Ag) screen; emission peak: 450 nm; slow ZnS(Ag) scintillation component	Thermal neutron-sensitive screen	Converts thermal neutrons through the ^6^Li(n,α)^3^H reaction and produces slower scintillation signals separable from the plastic-scintillator response.

^a^ For EJ276, approximate mean decay times of the first three components are 13, 35, and 270 ns for γ induced pulses and 13, 59, and 460 ns for neutron-induced pulses [16]. The material properties and reasons for selection were compiled from manufacturer datasheets and related studies [6,7,13,16,17,18,19,20,21,22].

**Table 2:** Main instruments and auxiliary materials used in this work.

Item	Model/Specification	Manufacturer/Country	Function
Photomultiplier tube	XP3232	Hainan Zhanchuang Photonics Technology Co., Ltd., Chengmai, Hainan, China	Scintillation-light readout.
Digital oscilloscope	HDO4104A, 1 GHz, 10 GS/s	Teledyne LeCroy, Chestnut Ridge, NY, USA	Waveform acquisition.
Digitizer	CAEN DT5751, 500 MHz, 1 GS/s	CAEN S.p.A., Viareggio, Italy	PMT gain and SPE calibration.
Optical silicone grease	SL612	Beijing Hoton Technology Co., Ltd., Beijing, China	Optical coupling between scintillator and PMT.
PMMA plates	Transparent plates with a 6.1 cm × 6.1 cm opening	local commercial supplier, Guangzhou, China	Mechanical protection and support for EJ426.
HDPE moderator	1–3 cm thickness	local commercial supplier, Guangzhou, China	Neutron moderation in Am–Be measurements.
Lead bricks	Commercial lead shielding bricks	local commercial supplier, Guangzhou, China	Reduction of environmental γ ray background.

Manufacturer and country information is provided to improve experimental reproducibility. Technical information was compiled from manufacturer datasheets and related references [13,24,25,26].

**Table 3:** γ ray emission energies and corresponding Compton edge energies of the three calibration sources.

Gamma Ray Source	$E_{\gamma }$ (MeV)	$E_{C}$ (MeV)
^137^Cs	0.662	0.477
^22^Na	0.511; 1.275	0.341; 1.062
^60^Co	1.173; 1.333	0.963; 1.118

The γ ray emission energies were taken from standard nuclear decay data [27]. The Compton edge energies were calculated using Equation (2) [5].

**Table 4:** PSD fitting results and FOM values for the EJ276+EJ426 assembly under different HDPE moderator thicknesses.

HDPE	μ Values (γ,f.n.,t.n.)	${\mathrm{FOM}}_{f.n./\gamma }$	${\mathrm{FOM}}_{t.n./f.n.}$	${\mathrm{FOM}}_{t.n./\gamma }$
1 cm	0.1071, 0.1799, 0.6237	0.8175±0.0040	3.7625±0.1632	5.0283±0.2491
3 cm	0.1037, 0.1783, 0.6181	0.9493±0.0040	4.3986±0.0771	6.1240±0.1265

Here, f.n. and t.n. denote fast neutron and thermal neutron events, respectively. The peak positions are listed in the order of γ, f.n., and t.n. FOM values were calculated using Equation (5).

**Table 5:** Event counts and relative fractions of the three PSD components for the EJ276+EJ426 assembly under different HDPE moderator thicknesses. The data were acquired over 10 min.

HDPE Thickness	Thermal Neutrons	Fast Neutrons	γ Rays	Total Event Count
1 cm	674±26 (0.463±0.018)%	65,911±257 (45.29±0.13)%	78,944±281 (54.25±0.13)%	145,529
2 cm	709±27 (0.541±0.020)%	55,122±235 (42.03±0.14)%	75,311±274 (57.43±0.14)%	131,142
3 cm	672±26 (0.568±0.022)%	46,135±215 (38.99±0.14)%	71,521±267 (60.44±0.14)%	118,328

The count uncertainties were estimated from Poisson statistics as $\sigma_{N}=\sqrt{N}$. The fraction uncertainties were estimated from binomial statistics as $\sigma_{p}=\sqrt{p\left(1-p\right)/N_{\mathrm{total}}}$, where $p=N_{i}/N_{\mathrm{total}}$. Only statistical uncertainties are reported.

## Data Availability

The data supporting the findings of this study are available from the corresponding author upon reasonable request.
